# Supplementary material for: Perceptography unveils the causal contribution of inferior temporal cortex to visual perception
Source: Nat Commun. 2024 Apr 18;15:3347. doi: 10.1038/s41467-024-47356-8 (PMC11026389; doi:10.1038/s41467-024-47356-8)
Supplement: Supplementary file 1 — Supplementary Information [file 41467_2024_47356_MOESM1_ESM.pdf]

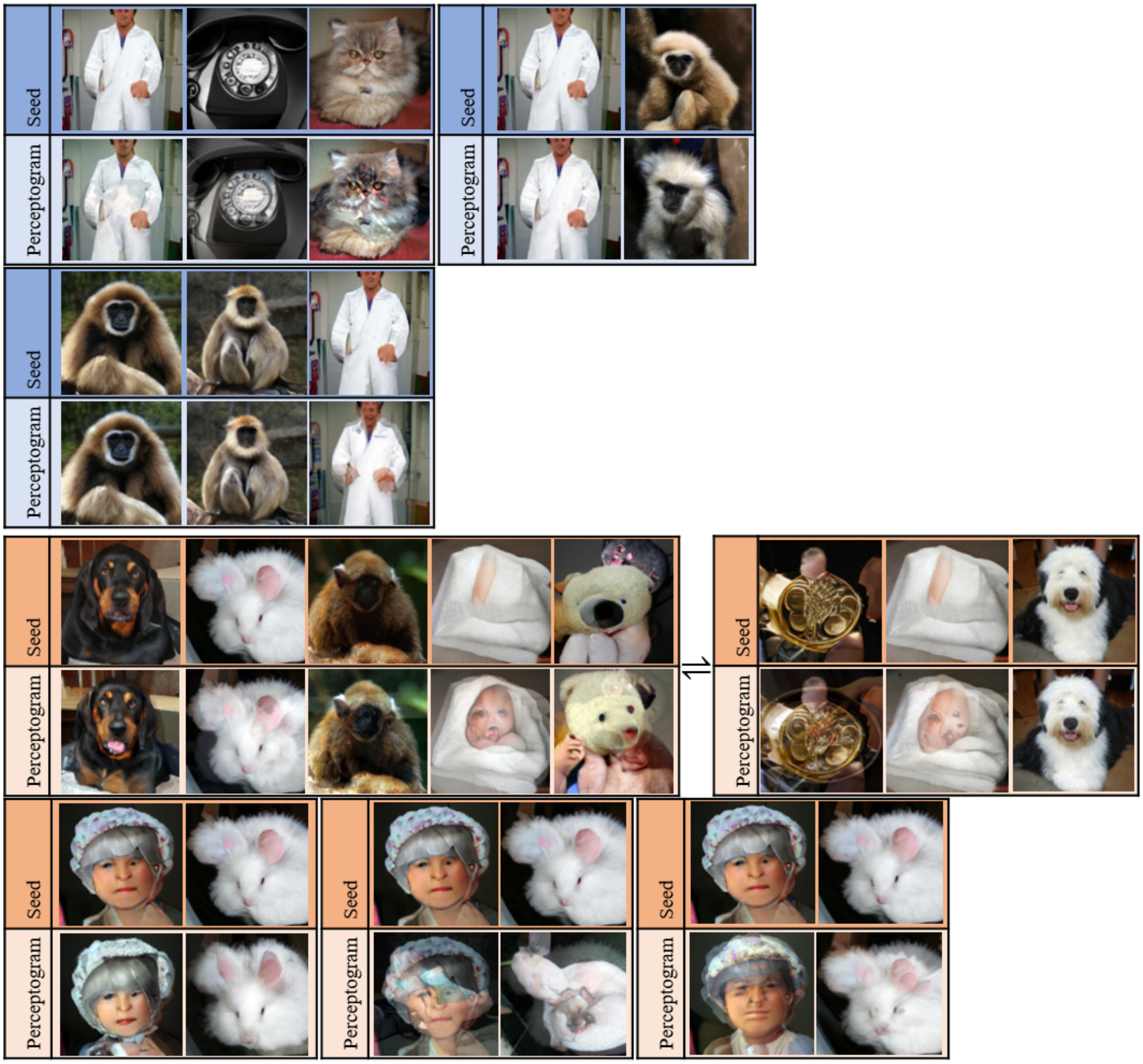


**Supplementary Fig 1. Perceptograms**. The rectangular boxes surrounding groups of images indicate the perceptograms obtained from the same cortical position using different seed images for the high-intensity stimulation condition. For three seed images, in monkey Ph, we tried even higher stimulation intensity. The "⇌" sign shows the blocks from the same cortical position but different stimulation intensities. Please note that subtle changes from the seed image are sometimes hard to see in these small static images, but they are far more visible when the seed image and the perceptogram are viewed in temporal sequences, such as in the experiment. We did not include the low-intensity perceptograms here because the changes were too subtle and difficult to notice in the image thumbnails. Blue: Sp, Orange: Ph

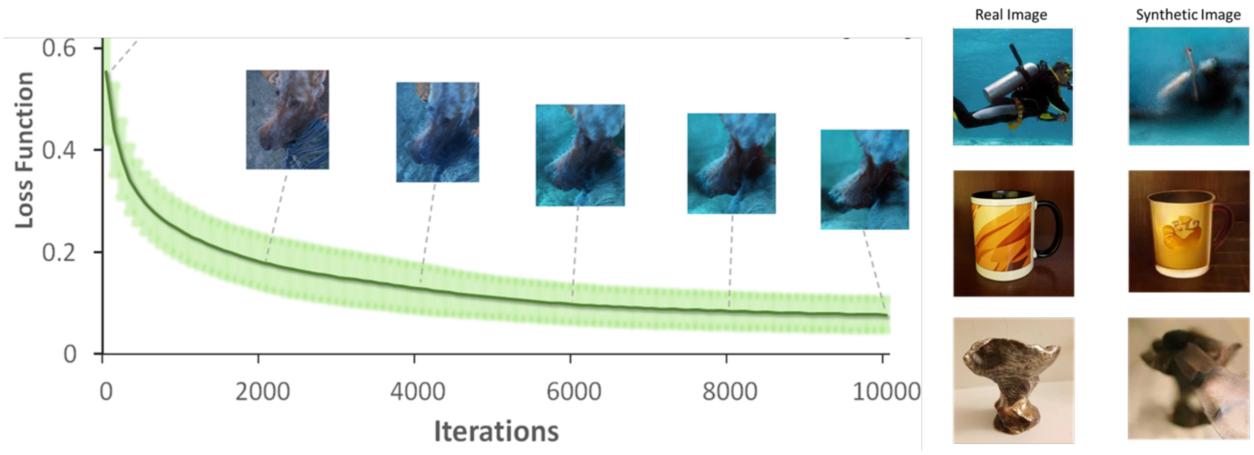


**Supplementary Fig 2: Recreating random images with Ahab and Davinci.** To test the quality of Ahab optimization and Davinci image illustration, seven random images (three shown here) \ that were not in the ImageNet (BigGAN and Davinci pretrained sets) dataset were selected. Ahab was then used to optimize a random Davinci image to be as close to the target as possible. The abscissa shows the loss function (pixel distance), and the ordinate represents Ahab iteration cycles. The shaded green shows ±1 standard error of the mean. Examples of images are shown; natural images in the left column and Ahab-optimized images in the right column.

Scuba diver image reproduced under a Attribution-NonCommercial 4.0 International (CC BY-NC 4.0)
https://creativecommons.org/licenses/by-nc/4.0/deed.en
